# Postpartum Depression and Anxiety in COVID-19-Positive and COVID-19-Negative Mothers: Insights From a Dedicated Hospital in Eastern India

**DOI:** 10.7759/cureus.80753

**Published:** 2025-03-18

**Authors:** Monika Anant, Priyanka Raj, Sangam Jha, Rajeev Ranjan, Samshad Ahmad, Chandni Sinha, Som Prabh, Sonam Yadav

**Affiliations:** 1 Obstetrics and Gynecology, All India Institute of Medical Sciences, Patna, IND; 2 Obstetrics, All India Institute of Medical Sciences, Patna, IND; 3 Psychiatry, All India Institute of Medical Sciences, Patna, IND; 4 Community and Family Medicine, All India Institute of Medical Sciences, Patna, IND; 5 Anesthesia, All India Institute of Medical Sciences, Patna, IND; 6 Obstetrics and Gynecology, All India Institute of Medical Sciences, New Delhi, IND

**Keywords:** covid, depression, depression scales, postpartum, stress

## Abstract

Objective

Women experienced mental health issues during pregnancy and postpartum, with the prevalence of depression and anxiety varying across different regions during the pandemic. A study was conducted to evaluate the symptoms of postpartum depression (PPD) and anxiety in women who tested positive and negative for COVID-19 and delivered in a tertiary-level hospital in Eastern India during the COVID-19 pandemic from 2020 to 2021. The objective was to explore the clinical and socio-demographic risk factors associated with PPD.

Methodology

A questionnaire-based cross-sectional study was conducted among women who were either positive or negative for COVID-19 in the Department of Obstetrics and Gynecology at All India Institute of Medical Sciences (AIIMS), Patna. A semi-structured questionnaire (the Edinburgh Postnatal Depression Scale (EPDS) questionnaire validated in Hindi) was used to collect socio-demographic and clinical details. The questionnaire included sections on socio-demographic characteristics, knowledge, attitudes, and behaviors related to COVID-19.

For the study, the EPDS score was calculated to assess feelings of the last seven days. A score below 8 was indicative of depression not likely, while scores of 9-11 indicated a possibility of depression, and scores of 12-13 suggested a high likelihood of depression. The cut-off score of 12 or higher was used to compare group differences in depression. The anxiety dimension was measured as the cumulative score from items 3, 4, and 5 in the EPDS (EPDS-3A). The sample size was determined to be 51 in each group, assuming a threefold increase in PPD among COVID-19-positive women who delivered at AIIMS Patna, with a study power of 80% and a significance level of 5%.

Results

A total of 327 candidates were invited to participate, of which 290 completed questionnaires were analyzed, comprising 237 COVID-19-negative and 53 COVID-19-positive mothers.

The mean ages, age group distribution, family structure, and residence type were similar in both COVID-19-positive and COVID-19-negative mothers. The prevalence of depression among COVID-19-negative mothers was 13.5% (32/237) with a mean EPDS score of 5.4 ± 3.8 as compared to 39.6% (21/53) with a mean EPDS score of 11.7 ± 3.3 among COVID-19-positive mothers.

A statistically significant association of PPD was noted with financial crisis (59.4%) in COVID-19-negative mothers. Poor family support was associated with both COVID-19-negative (81.2%) and COVID-19-positive (66.7%) mothers. Poor availability of medical services (66.7%), societal discrimination (76.2%), and loss of/minimal leisure activities (81%) were significantly associated with COVID-19-positive mothers.

Financial crisis (adjusted odds ratio (AOR): 4.3; 95% CI: 1.76-10.38; p = 0.001) and poor family support (AOR: 4.1; 95% CI: 1.33-12.29; p = 0.01) emerged as independent predictors of depression among COVID-19-negative mothers. Among COVID-19 positives, illiteracy (AOR: 2.3; 95% CI: 1.5-9.2; p = 0.01) and social discrimination (AOR: 16.5; 95% CI: 1.9-144.2; p = 0.01) were the independent predictors for PPD.

Conclusions

The prevalence of PPD and anxiety was found to be three times higher in COVID-19-positive mothers. Significant contributing factors included poor family support, lack of access to antenatal services, societal discrimination, and limited leisure activities during the pandemic. Low literacy and societal discrimination emerged as key predictors of PPD.

## Introduction

Pregnant and postpartum women undergo rapid physical and psychological transitions, making them particularly vulnerable to mental health disorders. According to the American College of Obstetricians and Gynecologists, depression and anxiety affect approximately 12-13% (one in seven) of women during the perinatal period [1]. These conditions have been linked to an increased risk of preterm delivery during pregnancy, impaired mother-infant bonding postpartum, and delayed cognitive and emotional development in children. Additionally, the prevalence of postnatal depression is higher in low- and middle-income countries compared to high-income nations [2].

The emergence of the novel severe acute respiratory syndrome coronavirus 2 (SARS-CoV-2) led to unprecedented restrictions on human activities. Fear of the disease, its high mortality rate, limited healthcare access, patient isolation, and reduced physical contact with loved ones further exacerbated psychological distress. Studies have reported a significantly higher prevalence of mental health issues across all populations during the pandemic [3].

Anxiety disorders in postpartum women can range from mild conditions such as generalized anxiety to more severe forms, including panic attacks, obsessive-compulsive disorder, post-traumatic stress disorder, and extreme fear of childbirth [4,5]. Women with a personal or family history of mental illness are at a higher risk of developing postpartum mental health issues.

Despite its significant impact, mental health assessment remains a neglected area, with less than half of affected women being diagnosed or treated during and after pregnancy, particularly in low-income countries.

The COVID-19 pandemic further worsened maternal mental health by restricting access to diagnosis and treatment, contributing to higher depression rates among pregnant women [6].

This study aimed to evaluate postpartum depression (PPD) and anxiety symptoms in COVID-19-positive and COVID-19-negative women and to determine the prevalence of PPD and identify the clinical and socio-demographic risk factors associated with PPD during this period.

## Materials and methods

The study was conducted in the Department of Obstetrics and Gynecology at All India Institute of Medical Sciences (AIIMS), Patna, after obtaining approval from the institute's Institutional Research Cell (approval number: AIIMS/Pat/IRC/2020/572). It included postpartum women, both COVID-19 positive and COVID-19 negative, who were tested using SARS-CoV-2 reverse transcription-polymerase chain reaction (RT-PCR).

Sample selection

Postpartum women who delivered or were admitted for medical or obstetrical reasons and provided informed consent were included in the study. Exclusion criteria included mothers with known psychiatric illnesses, cognitive impairment due to mental retardation or brain injury/syndrome, or chronic medical or surgical conditions that could affect the reliability of information provided. Women with alcohol or drug use disorders in the past six months, those experiencing major life stressors (such as the death of a family member, divorce, or separation from a partner), or those with medical or surgical complications related to delivery were also excluded. Additionally, mothers of infants with congenital anomalies or those who experienced intrauterine fetal death were not approached for participation. 

The sample size was determined to be 51 in each group, based on an assumed threefold increase in PPD among COVID-19-positive women, with a study power of 80% and a significance level of 5%.

Test measures

A questionnaire-based cross-sectional study was conducted from March 2020 to February 2021 on COVID-19-positive and COVID-19-negative postpartum women. Written informed consent was obtained from all participants. A semi-structured questionnaire was used to collect socio-demographic and clinical details, including the following: (A) socio-demographic characteristics, such as age, religion, occupation, education, residential address, previous clinical diagnoses, investigations performed, and psycho-social-clinical factors, and (B) the Edinburgh Postnatal Depression Scale (EPDS) which is a 10-item screening tool used to assess depression and anxiety in postpartum women. A validated Hindi version of the EPDS was administered. 

For this study, the EPDS evaluated participants' feelings over the past seven days. A cutoff score of 12 or higher was used to detect possible depression. Scores were categorized as follows: <8 (depression unlikely), 9-11 (possible depression), and 12-13 (high likelihood of depression).

The anxiety dimension was assessed using the cumulative score from items 3, 4, and 5 of the EPDS (EPDS-3A). Data analysis was conducted to compare PPD and anxiety between the two groups. 

The data collection was carried out by trained residents and nursing officers posted in COVID-19 wards. They received training from the principal investigator on quality control, data completeness, and research ethics. All completed questionnaires were reviewed for accuracy and consistency. The collected data was then cleaned, coded, and entered into MS Excel (Microsoft Corp., Redmond, WA, USA) for further analysis.

Statistical calculation

EPDS scores were calculated, and participants were categorized as depressed (EPDS score ≥12) or not depressed (EPDS score <12). Age was grouped into three categories: <20 years, 20-30 years, and >30 years. 

Results for continuous variables (e.g., age) were presented as mean and standard deviation (SD), while categorical variables were expressed as frequencies and percentages. The chi-squared test was used to assess associations between categorical variables, with a p-value of <0.05 considered statistically significant. 

To identify independent predictors of PPD, multiple logistic regression analysis was performed. Variables that showed a significant association in the univariate analysis (p < 0.05) were included in the regression model for further analysis.

## Results

A total of 327 candidates were invited to participate in the study. Twenty-five women were excluded based on exclusion criteria or refusal to enroll, and 12 women returned incomplete questionnaires. 

The final analysis was conducted on 290 completed questionnaires, comprising 237 COVID-19-negative and 53 COVID-19-positive postpartum mothers.

COVID-19-negative mothers

More than half were in the 20-30-year age group (72.5%). The majority were literate (83.1%), were employed (55.9%), lived in nuclear families (60.1%), and resided in urban areas (77.5%). Among them, illiteracy (34.4%) and belonging to a nuclear family (77.4%) were found to be statistically associated with depression. Most COVID-19-negative mothers were primigravida (46.4%) and nulliparous (55.3%). Approximately half had a normal vaginal delivery (52.7%) and delivered a male child (57.4%). However, no statistically significant difference was observed between depressed and non-depressed mothers concerning obstetric history (Table 1).

**Table 1:** Socio-demographic characteristics of COVID-19-negative mothers *Significant SD: standard deviation; LSCS: lower segment cesarean section; NVD: normal vaginal delivery

Characteristics		Not depressed (score <12), n = 205 (100)	Depressed (score >12), n = 32 (100)	P-value
Age (years)	Mean (SD)	25.6 (3.6)	25.5 (4.1)	
	<20	24 (11.7)	2 (6.3)	0.475
	20-30	146 (71.2)	26 (81.2)	
	>30	35 (17.1)	4 (12.5)	
Education	Illiterate	29 (14.2)	11 (34.4)	0.004*
	Literate	176 (85.8)	21 (65.6)	
Occupation	Housewife	89 (43.6)	15 (46.9)	0.731
	Working	115 (56.4)	17 (53.1)	
Family	Nuclear	116 (57.4)	24 (77.4)	0.034*
	Joint	86 (42.6)	7 (22.6)	
Locality	Rural	45 (22.1)	8 (25)	0.71
	Urban	159 (77.9)	24 (75)	
Gravida	1	94 (45.8)	16 (50)	0.08
	2	58 (28.3)	5 (15.6)	
	3	33 (16.1)	10 (31.3)	
	>3	20 (9.8)	1 (3.1)	
Parity	Nulliparous	115 (56.1)	16 (50)	0.01*
	Multiparous	90 (43.9)	16 (50)	
Mode of delivery	LSCS	102 (49.7)	10 (31.3)	0.05*
	NVD	103 (50.3)	22 (68.7)	
Gender of baby	Female	87 (42.4)	14 (43.7)	0.89
	Male	118 (57.6)	18 (56.3)	

COVID-19-positive mothers

The majority were aged 20-30 years (60.4%), literate (73.6%), housewives (79.3%), from joint families (58.5%), and residing in urban areas (64.7%). Similar to COVID-19-negative mothers, a statistically significant association was found between depression and both illiteracy (52.4%) and living in a nuclear family (52.4%). Most were primigravida (46.2%) and nulliparous (54.9%). A higher proportion underwent cesarean sections (78.9%) and delivered female children (56.6%). Notably, depression rates were significantly higher among those who had a cesarean delivery and those who gave birth to female children (Table 2).

**Table 2:** Socio-demographic characteristics of COVID-19-positive mothers *Significant SD: standard deviation; LSCS: lower segment cesarean section; NVD: normal vaginal delivery

Characteristics		Not depressed (score <12), n = 32 (100)	Depressed (score >12), n = 21 (100)	P-value
Age (years)	Mean (SD)	26.8 (5.8)	26.9 (4.8)	
	<20	5 (15.6)	4 (19.1)	0.94
	20-30	20 (62.5)	12 (57.1)	
	>30	7 (21.9)	5 (23.8)	
Education	Illiterate	3 (9.4)	11 (52.4)	0.01*
	Literate	29 (90.6)	10 (47.6)	
Occupation	Housewife	26 (81.3)	16 (76.2)	0.657
	Working	6 (18. 7)	5 (23.8)	
Family	Nuclear	11 (34.4)	11 (52.4)	0.034*
	Joint	21 (65.6)	10 (47.6)	
Locality	Rural	12 (37.5)	6 (28.6)	0.401
	Urban	20 (62.5)	15 (71.4)	
Gravida	1	16 (50)	8 (38.1)	0.34
	2	10 (31.3)	5 (23.9)	
	3	3 (9.4)	4 (19)	
	>3	2 (6.3)	4 (19)	
Parity	Nulliparous	20 (62.5)	9 (42.9)	0.331
	Multiparous	12 (37.5)	12 (57.1)	
Mode of delivery	LSCS	26 (81.25)	15 (71.4)	0.05*
	NVD	6 (18.75)	6 (28.6)	
Gender of baby	Female	15 (46.9)	15 (71.4)	0.05*
	Male	17 (53.1)	6 (28.6)	

The mean age, age group distribution, family structure, and residence type were similar in both COVID-19-positive and COVID-19-negative mothers. However, the prevalence of depression among COVID-19-negative mothers was 15.6% (32/327) with a mean EPDS score of 5.4 ± 3.8, whereas among COVID-19-positive mothers, it was significantly higher at 39.6% (21/53), with a mean EPDS score of 11.7 ± 3.3.

Among COVID-19-negative mothers, the majority of both depressed (33.3%) and non-depressed (34.4%) mothers had undergone at least three antenatal check-ups. However, a significant association was found between COVID-19-positive status and fewer antenatal visits. Medical conditions and antenatal complications were more prevalent among COVID-19-positive women, though the difference was not statistically significant when comparing depressed and non-depressed mothers (Table 3).

**Table 3:** Relevant antenatal and medical history of COVID-19-negative mothers and COVID-19-positive mothers *Significant

		COVID-19-negative mothers			COVID-19-positive mothers		
Characteristics		Not depressed (score <12)	Depressed (score >12)	P-value	Not depressed (score <12)	Depressed (score >12)	P-value
Number of antenatal check-ups	1	18 (8.8)	13 (40.6)	0.527	1 (3.2)	1 (4.8)	0.05*
	2	60 (29.3)	10 (31.3)		11 (34.4)	7 (33.3)	
	3	99 (48.3)	5 (15.6)		16 (50)	9 (42.8)	
	>4	28 (13.7)	4 (12.5)		3 (9.4)	4 (19)	
Antenatal complication	No	118 (57.6)	24 (75)	0.298	3 (9.4)	1 (5)	0.06
	Yes	87 (42.4)	8 (25)		29 (90.6)	20 (95.2)	
Medical disease	No	167 (81.5)	25 (78.1)	0.366	22 (68.75)	12 (57.1)	0.6
	Yes	38 (18.5)	7 (21.9)		10 (31.25)	9 (42.9)	
Total		205 (100)	32 (100)		32 (100)	21 (100)	

A statistically significant association was observed between financial crises (59.4%) and depression among COVID-19-negative mothers. Additionally, poor family support was strongly linked to depression in both COVID-19-negative (81.2%) and COVID-19-positive (66.7%) mothers (Table 4).

**Table 4:** Distribution of COVID-19-negative and COVID-19-positive mothers according to the financial crisis and family support *Significant

Characteristics	COVID-19-negative mothers			COVID-19-positive mothers		
	Not depressed (score <12)	Depressed (score >12)	P-value	Not depressed (score <12)	Depressed (score >12)	P-value
Financial crisis						
No	162 (79.1)	13 (40.6)	0.03*	3 (9.4)	5 (23.8)	0.23
Yes	43 (20.9)	19 (59.4)		29 (90.6)	16 (76.2)	
Poor family support						
No	90 (43.9)	6 (18.8)	0.04*	25 (78.1)	7 (33.3)	0.03*
Yes	115 (56.1)	26 (81.2)		7 (21.9)	14 (66.7)	
Total	205 (100)	32 (100)		32 (100)	21 (100)	

Among COVID-19-positive mothers, poor availability of medical and antenatal services (66.7%), societal discrimination (76.2%), and loss of or minimal leisure activities (81%) were significantly associated with depression (Table 5).

**Table 5:** Distribution of COVID-19-positive mothers as per their situation due to COVID-19 status *Significant

Characteristics		Not depressed (score <12)	Depressed (score >12)	P-value
Poor availability of medical services/antenatal services	No	22 (68.8)	8 (38.1)	0.02*
	Yes	10 (31.3)	14 (66.7)	
Poor provision of psychological first aid	No	3 (9.4)	2 (9.5)	0.98
	Yes	29 (90.6)	19 (90.5)	
Societal discrimination	No	22 (68.8)	5 (23.8)	0.001*
	Yes	10 (31.3)	16 (76.2)	
Loss of/minimal leisure activities	No	15 (46.9)	4 (19)	0.03*
	Yes	17 (53.1)	17 (81)	
Print and social media mis-/overinformation	No	16 (50)	12 (57.1)	0.6
	Yes	16 (50)	9 (42.9)	
Hospital staff discrimination	No	26 (81.3)	17 (80.9)	0.163
	Yes	6 (18.7)	4 (19.1)	
Difficulties in accessing grocery and daily food items	No	20 (62.5)	15 (71.4)	0.502
	Yes	12 (37.5)	6 (28.6)	
Total		32 (100)	21 (100)	

Multiple logistic regression analysis identified financial crises and poor family support as independent predictors of depression among COVID-19-negative mothers. In contrast, illiteracy and social discrimination were the key independent predictors of depression among COVID-19-positive mothers (Table 6).

**Table 6:** Predictors of depression among COVID-19-positive and COVID-19-negative mothers *Significant; **highly significant AOR: adjusted odds ratio; CI: confidence interval; LSCS: lower segment cesarean section; NVD: normal vaginal delivery

Characteristics	COVID-19-negative mothers		COVID-19-positive mothers	
	AOR (95% CI)	P-value	AOR (95% CI)	P-value
Education				
Illiterate	1	0.14	1	0.001*
Literate	0.45 (0.15-1.35)		0.05 (0.01-0.48)	
Family				
Nuclear	1	0.41	1	0.9
Joint	0.56 (0.18-1.74)		0.92 (0.14-5.9)	
Mode of delivery				
LSCS	1	0.18	-	-
NVD	1.6 (0.62-4.12)		-	
Gender of baby				
Female	-	-	1	-
Male	-		0.15 (0.02-1.1)	
Parity				
Nulliparous	1	0.27	-	-
Multiparous	1.2 (0.88-1.58)		-	
Financial crisis	4.3 (1.76-10.38)	0.001**	-	-
Poor family support	4.1 (1.33-12.29)	0.01*	1.1 (0.15-8.4)	0.91
Poor availability of medical services/antenatal services	-	-	5.6 (0.79-39.4)	0.085
Social discrimination	-	-	16.5 (1.9-144.2)	0.01*
Print/social media misinformation	-	-	0.63 (0.09-4.1)	0.63
Loss of/minimal leisure activity	-	-	4.9 (0.68-35.7)	0.11

## Discussion

The study examined the prevalence of PPD and its predictors among mothers who delivered in a dedicated COVID-19 hospital during the pandemic. The prevalence of PPD among COVID-19-positive mothers was found to be 39.6%, significantly higher than PPD rates under non-pandemic conditions. 

During the pandemic, studies from China estimated PPD prevalence six weeks post-delivery to range from 11.8% to 30% at the peak of COVID-19 [7,8]. However, our findings align with reports from other Asian countries, where PPD prevalence has been reported between 3.5% and 63.3% [9]. Additionally, a cross-sectional study in Brazil found a similar PPD prevalence of 27.9% among low-income women [10].

The higher rates of PPD observed in this study may be attributed to lockdown measures implemented to contain COVID-19, reduced communication with friends and family, fear of infection, job losses, and economic instability. The prolonged duration of the pandemic and associated prevention and control measures further contributed to the increased incidence. 

COVID-19-positive postpartum women had significantly higher EPDS scores, indicating increased depressive symptoms. A significant association between illiteracy and depression was observed in both COVID-19-positive and COVID-19-negative groups, likely due to a lack of understanding of the disease. Existing studies examining the relationship between low literacy and psychiatric symptoms are largely cross-sectional and limited in establishing causality. Future interventions for mental health will depend on a deeper understanding of the causal link between illiteracy and depression [11].

Gazmararian et al. found that low health literacy tripled depression risk, though it ceased to be an independent factor after adjusting for demographics and social support [12]. This study also linked higher depression rates to nuclear families, where limited support may heighten postpartum stress. Prince et al. similarly found that social support deficits strongly associated with depression in the elderly [13]. Additionally, emergency cesarean sections correlated with higher EPDS scores, likely due to unpreparedness and restricted postnatal support during COVID-19. Ilska et al. previously reported increased PPD risk following emergency cesareans [14], with symptoms appearing earlier and more severe than in elective or vaginal deliveries.

Financial instability during COVID-19, due to widespread job losses, was significantly associated with depression among postnatal women (59.4%). Economic hardships inevitably impact mental health. Poor family support was also linked to depression in both groups (66.7%), consistent with findings from a Chinese study by Liang et al. (November 2020) [8]. In India, family support plays a crucial role in helping postpartum women navigate challenges. A well-established link exists between low family support and depression, as it buffers against adverse life circumstances. Poor family support increases the risk of PPD by 4.2 times, while financial crises raise it by 4.1 times.

Poor availability of medical/antenatal services (66.7%), societal discrimination (76.2%), and minimal leisure activities (81%) were significantly linked to depression among COVID-19-positive mothers. A study found a 2.4 times higher odds of PPD in women with low education and perceived discrimination, while higher education served as a protective factor [15]. Although medical conditions and antenatal complications were more common in COVID-19-positive women, they were not significantly different between depressed and non-depressed groups. Conditions like diabetes and respiratory and cardiac diseases increased the risk of severe COVID-19, an expected outcome [16]. While numerous studies focus on COVID-19-positive PPD mothers, this study highlights the high prevalence of depression even among COVID-19-negative mothers, with financial adversity and poor familial support as key contributors. COVID-19-positive postpartum women with low literacy, financial instability, limited healthcare access, reduced leisure activities, and societal discrimination were at greater risk of depression. Notably, those facing discrimination for their COVID-19 status had 16.5 times higher odds of developing PPD. A recent Chinese meta-analysis also linked family issues and lockdown policies to increased PPD prevalence [17].

The pandemic affected all postpartum women, heightening anxiety, stress, and depression during antenatal and postnatal periods. Future studies should explore the long-term impact of PPD on mothers and children with a longitudinal study design, include a more diverse sample by multicentric centre, and explore interventions for affected mothers to improve mental health outcomes.

## Conclusions

The prevalence of PPD and anxiety is almost three times higher in COVID-19-positive mothers, largely due to poor family support, unavailability of medical services/antenatal services, societal discrimination, and minimal leisure activities. Predictors of PPD are low literacy and societal discrimination for which efforts from family and society need to be improved manifold. 

## References

[1] (2018). ACOG Committee opinion no. 757: screening for perinatal depression. Obstet Gynecol.

[2] Parsons CE, Young KS, Rochat TJ, Kringelbach ML, Stein A (2012). Postnatal depression and its effects on child development: a review of evidence from low- and middle-income countries. Br Med Bull.

[3] Wang C, Pan R, Wan X (2020). A longitudinal study on the mental health of general population during the COVID-19 epidemic in China. Brain Behav Immun.

[4] D'Souza R (2022). Antenatal and postnatal mental health: clinical management and service guidance. Best Pract Res Clin Obstet Gynaecol.

[5] Kumar R (1994). Postnatal mental illness: a transcultural perspective. Soc Psychiatry Psychiatr Epidemiol.

[6] Wu Y, Zhang C, Liu H (2020). Perinatal depressive and anxiety symptoms of pregnant women during the coronavirus disease 2019 outbreak in China. Am J Obstet Gynecol.

[7] Ding G, Niu L, Vinturache A (2020). "Doing the month" and postpartum depression among Chinese women: a Shanghai prospective cohort study. Women Birth.

[8] Liang P, Wang Y, Shi S, Liu Y, Xiong R (2020). Prevalence and factors associated with postpartum depression during the COVID-19 pandemic among women in Guangzhou, China: a cross-sectional study. BMC Psychiatry.

[9] Klainin P, Arthur DG (2009). Postpartum depression in Asian cultures: a literature review. Int J Nurs Stud.

[10] Faisal-Cury A, Menezes PR, d'Oliveira AF, Schraiber LB, Lopes CS (2013). Temporal relationship between intimate partner violence and postpartum depression in a sample of low income women. Matern Child Health J.

[11] Wolf MS, Gazmararian JA, Baker DW (2005). Health literacy and functional health status among older adults. Arch Intern Med.

[12] Gazmararian J, Baker D, Parker R, Blazer DG (2000). A multivariate analysis of factors associated with depression: evaluating the role of health literacy as a potential contributor. Arch Intern Med.

[13] Prince MJ, Harwood RH, Blizard RA, Thomas A, Mann AH (1997). Social support deficits, loneliness and life events as risk factors for depression in old age. The Gospel Oak Project VI. Psychol Med.

[14] Ilska M, Banaś E, Gregor K, Brandt-Salmeri A, Ilski A, Cnota W (2020). Vaginal delivery or caesarean section - severity of early symptoms of postpartum depression and assessment of pain in Polish women in the early puerperium. Midwifery.

[15] Stepanikova I, Kukla L (2017). Is perceived discrimination in pregnancy prospectively linked to postpartum depression? Exploring the role of education. Matern Child Health J.

[16] Conde-Agudelo A, Romero R (2022). SARS-CoV-2 infection during pregnancy and risk of preeclampsia: a systematic review and meta-analysis. Am J Obstet Gynecol.

[17] Lin C, Chen B, Yang Y (2023). Association between depressive symptoms in the postpartum period and COVID-19: a meta-analysis. J Affect Disord.

